# Tetra­aqua­bis(1,10-phenanthroline)bis­[μ_2_-1*H*-pyrazole-3,5-dicarboxyl­ato(3−)]tricopper(II) dihydrate

**DOI:** 10.1107/S1600536810012833

**Published:** 2010-04-24

**Authors:** Zhi-Gang Li, Shao-Ai Li, De-Quan Liu, Long He, Jing-Wei Xu

**Affiliations:** a Shenzhen Environmental Monitoring Center, Shenzhen 518008, People’s Republic of China; b State Key Laboratory of Electroanalytical Chemistry, Changchun Institute of Applied Chemistry, Chinese Academy of Sciences, Changchun 130022, People’s Republic of China

## Abstract

The title compound, [Cu_3_(C_5_HN_2_O_4_)_2_(C_12_H_8_N_2_)_2_(H_2_O)_4_]·2H_2_O,  is a trinuclear copper(II) complex in which two centrosymmetrically related pyrazole-3,5-dicarboxyl­ate(3−) and 1,10-phenanthroline ligands bind three Cu^II^ atoms, with one Cu^II^ atom located on a center of symmetry. In each complex, there are four coordinated water mol­ecules and two solvent water mol­ecules, which participate in extensive hydrogen-bond patterns. These inter­actions, as well as π–π inter­actions between neighbouring 1,10-phenanthroline ligands [shortest atom-to-atom distance = 3.363 (3) Å], extend the crystal structure into a three-dimensional supra­molecular network.

## Related literature

For the potential applications of novel coordination architectures as new classes of materials, see: Kitagawa *et al.* (2004 ▶). The potential coordination sites of 3,5-pyrazoledicarboxylate are highly accessible to metal ions, see: Li (2005 ▶). However, divalent copper ions have rarely been coordinated with 3,5-pyrazoledicarboxylic acid at ambient temperature, see: King *et al.* (2003 ▶).
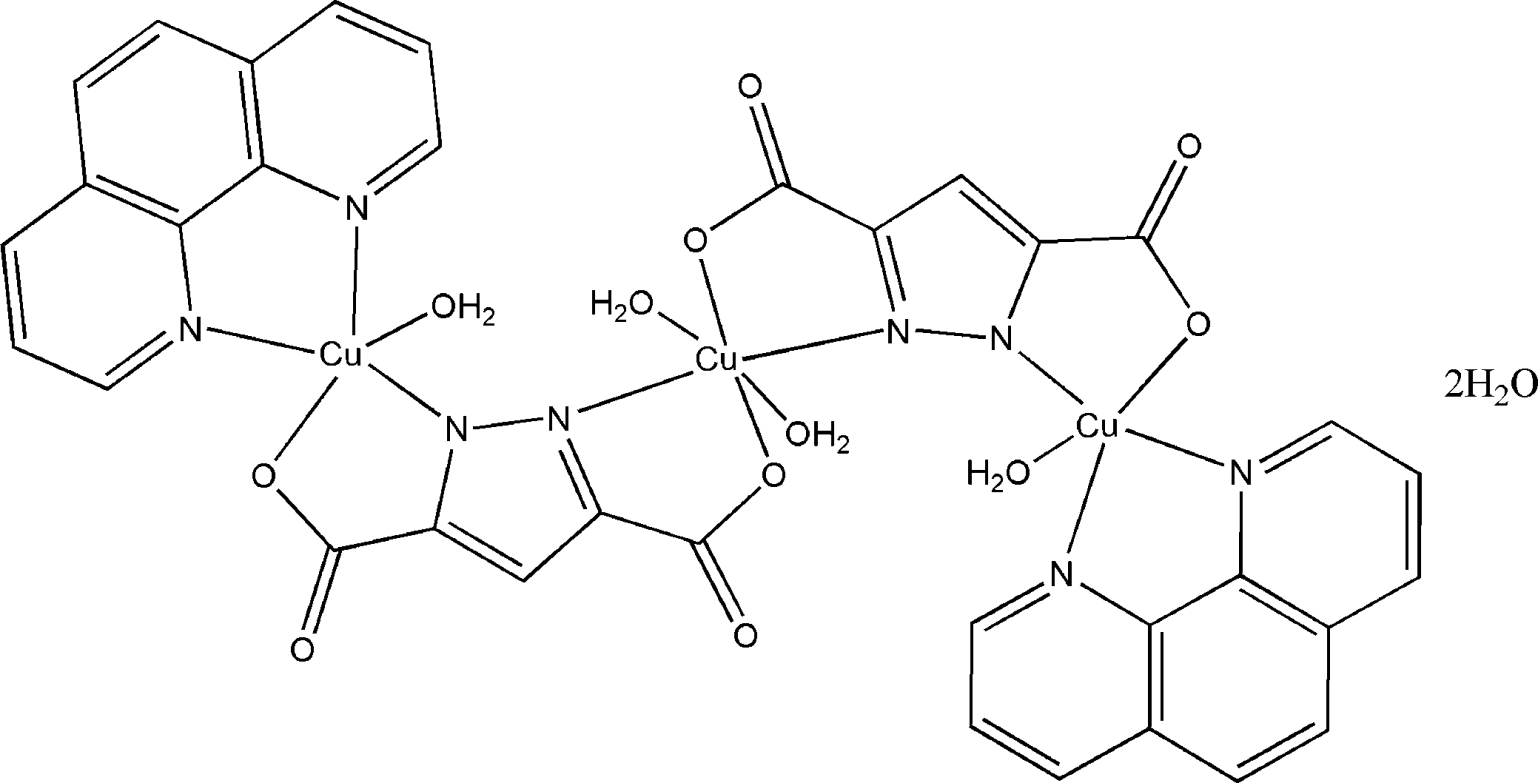


## Experimental

### 

#### Crystal data

[Cu_3_(C_5_HN_2_O_4_)_2_(C_12_H_8_N_2_)_2_(H_2_O)_4_]·2H_2_O
*M* *_r_* = 965.28
Triclinic, 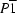

*a* = 7.7326 (8) Å
*b* = 9.3332 (9) Å
*c* = 12.6848 (12) Å
α = 100.204 (2)°
β = 98.376 (2)°
γ = 103.641 (2)°
*V* = 858.59 (15) Å^3^
*Z* = 1
Mo *K*α radiation
μ = 1.93 mm^−1^
*T* = 187 K
0.07 × 0.07 × 0.03 mm

#### Data collection

Bruker APEX CCD area-detector diffractometer
Absorption correction: multi-scan (*SADABS*; Bruker, 2003 ▶) *T*_min_ = 0.872, *T*_max_ = 0.937
4550 measured reflections
3151 independent reflections
2361 reflections with *I* > 2σ(*I*)
*R*_int_ = 0.039

#### Refinement

*R*[*F* ^2^ > 2σ(*F* ^2^)] = 0.071
*wR*(*F* ^2^) = 0.155
*S* = 1.10
3151 reflections
274 parameters
2 restraints
H atoms treated by a mixture of independent and constrained refinement
Δρ_max_ = 0.87 e Å^−3^
Δρ_min_ = −0.58 e Å^−3^

### 

Data collection: *SMART* (Bruker, 1998 ▶); cell refinement: *SAINT* (Bruker, 2003 ▶); data reduction: *SAINT*; program(s) used to solve structure: *SHELXS97* (Sheldrick, 2008 ▶); program(s) used to refine structure: *SHELXL97* (Sheldrick, 2008 ▶); molecular graphics: *SHELXTL* (Sheldrick, 2008 ▶); software used to prepare material for publication: *SHELXTL*.

## Supplementary Material

Crystal structure: contains datablocks global, I. DOI: 10.1107/S1600536810012833/ez2198sup1.cif

Structure factors: contains datablocks I. DOI: 10.1107/S1600536810012833/ez2198Isup2.hkl

Additional supplementary materials:  crystallographic information; 3D view; checkCIF report

## Figures and Tables

**Table 1:** Hydrogen-bond geometry (Å, °)

*D*—H⋯*A*	*D*—H	H⋯*A*	*D*⋯*A*	*D*—H⋯*A*
O5—H5*A*⋯O2^i^	0.87	2.05	2.842 (7)	152
O5—H5*B*⋯O4^ii^	0.90	1.95	2.824 (7)	163
O6—H6*A*⋯O4^i^	0.88 (5)	2.26 (6)	3.104 (8)	161 (7)
O6—H6*B*⋯O4^ii^	0.89 (3)	2.16 (3)	3.042 (8)	172 (10)
O7—H7*A*⋯O5^iii^	0.85	2.24	2.977 (8)	145
O7—H7*B*⋯O3^iv^	0.87	1.97	2.829 (7)	170

Symmetry codes: (i) 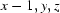; (ii) 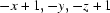; (iii) 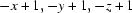; (iv) 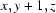.

**Fig. 1.**
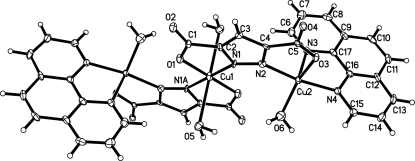
A view of (I), with the atom-labeling scheme and 30% probability displacement ellipsoids. [Symmetry code: (A) 1 - x, 1 - y, 1 - z.] For clarity the lattice water molecules have been omitted.

**Fig. 2.**
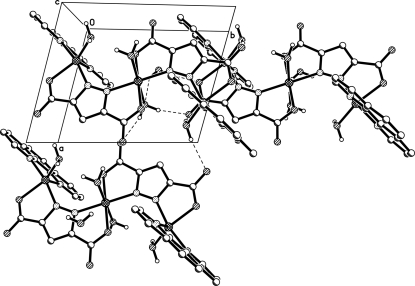
Perspective view of packing structure of (I) along the c axes, with hydrogen bonds indicated by dashed lines. For the sake of clarity, H atoms not involved in hydrogen bonds have been omitted.

## supplementary crystallographic information

### Comment

The design and synthesis of novel coordination architectures has attracted wide attention because of their intriguing network topologies and potential applications as new classes of materials (Kitagawa *et al.*, 2004). 3,5-pyrazoledicarboxylate has several potential coordination sites: both nitrogen atoms of the pyrazole ring and the four carboxylate oxygen atoms, which are highly accessible to metal ions (Li, 2005). However, divalent copper ions have rarely been coordinated with 3,5-pyrazoledicarboxylic acid under ambient temperatures (King *et al.*, 2003). In this study, we chose 3,5-pyrazoledicarboxylic acid and 1,10-phenanthroline as mixed ligands to obtain blue crystals of the title compound (I), which as shown in Fig. 1 is a copper(II) trimer.

The central copper atom, Cu(1), lies on a crystallographic inversion center, and has a six-coordinate octahedral geometry, in the which two oxygen atoms and two nitrogen atoms from two 3,5-pyrazoledicarboxylate ligands occupy the equatorial plane. The axial coordination sites are occupied by two water molecules. The Cu(1)—N/O bond distances span a very large range from 1.974 (5) to 2.595 (5) Å. The other two symmetry-related copper atoms, Cu(2), have pentacoordinate square-pyramidal geometry: a pyrazole nitrogen N(2) and a carboxylate oxygen O(3) from one 3,5-pyrazoledicarboxylato ligand occupy two coordination sites, two nitrogen atoms from one 1,10-phenanthroline chelate the Cu(2) atoms, while the remaining position is occupied by a water molecule. The Cu(2)—N/O bond distances range from 1.979 (6) to 2.171 (5) Å. The 3,5-pyrazoledicarboxylate ligand is nearly planar, with greatest deviation from the mean plane defined by the pyrazole ring by the carboxylate groups with values ranging from 0.0015 (1) to 0.0937 (1) Å. It can be seen that the ligand bite angles at the two different copper centers Cu(1) and Cu(2) are similar, 80.9 (3)° and 82.5 (4)°, respectively. This implies that the 3,5-pyrazoledicarboxylate ligand is a fairly rigid ligand and retains its integrity on metal chelation.

In the asymmetric unit, there is one lattice water molecule, O(7), and because each trimer contains four coordinated water molecules and carboxylate oxygen atoms, a complex network of hydrogen-bonding interactions is formed. Each 3,5-pyrazoledicarboxylato contains four hydrogen bond acceptors, while each coordinated water molecule acts as both a two hydrogen bond donor and a hydrogen bond acceptor, and the lattice water molecule is only a two hydrogen bond donor. In the crystal structure, π-π interactions also exist between neighbouring 1,10-phenanthroline ligands, with the nearest atom-to-atom distance between neighbouring 1,10-phenanthroline ligands being 3.363 (3) Å. The strong hydrogen bonding interactions as well as π-π interactions extend the crystal structure into a three-dimensional supramolecular network (Fig. 2).

### Experimental

The title complex was prepared by the addition of Cu(BF_4_)_2_ (20 mmol), 1,10-phenanthroline (30 mmol) and 3,5-pyrazoledicarboxylic acid (30 mmol) to 40 ml water. The mixture was stirred for 1 h, a blue precipitate was obtained. A minimum amount of ammonia (14 *M*) was added to give a blue solution. Suitable crystals were obtained after standing at room temperature for several days (yield 42% based on Cu).

### Refinement

H atoms were placed geometrically and refined with fixed individual displacement parameters [*U*_iso_(H) = 1.2*U*_eq_(C,*N*)], using a riding model, with a C—H distance of 0.93 Å. The H atoms bonded to O atoms of water molecules were located in a difference Fourier map and refined with fixed individual displacement parameters *U*_iso_(H) = 1.2*U*_eq_(O).

### Crystal data

**Table:**

[Cu_3_(C_5_HN_2_O_4_)_2_(C_12_H_8_N_2_)_2_(H_2_O)_4_]·2H_2_O	*Z* = 1
*M**_r_* = 965.28	*F*(000) = 489
Triclinic, *P*1	*D*_x_ = 1.867 Mg m^−^^3^
Hall symbol: -P 1	Mo *K*α radiation, λ = 0.71073 Å
*a* = 7.7326 (8) Å	Cell parameters from 737 reflections
*b* = 9.3332 (9) Å	θ = 2.3–22.4°
*c* = 12.6848 (12) Å	µ = 1.93 mm^−^^1^
α = 100.204 (2)°	*T* = 187 K
β = 98.376 (2)°	Block, blue
γ = 103.641 (2)°	0.07 × 0.07 × 0.03 mm
*V* = 858.59 (15) Å^3^	

### Data collection

**Table:**

Bruker APEX CCD area-detector diffractometer	3151 independent reflections
Radiation source: fine-focus sealed tube	2361 reflections with *I* > 2σ(*I*)
graphite	*R*_int_ = 0.039
φ and ω scans	θ_max_ = 25.5°, θ_min_ = 1.7°
Absorption correction: multi-scan (*SADABS*; Bruker, 2003)	*h* = −8→9
*T*_min_ = 0.872, *T*_max_ = 0.937	*k* = −11→9
4550 measured reflections	*l* = −9→15

### Refinement

**Table:**

Refinement on *F*^2^	Primary atom site location: structure-invariant direct methods
Least-squares matrix: full	Secondary atom site location: difference Fourier map
*R*[*F*^2^ > 2σ(*F*^2^)] = 0.071	Hydrogen site location: inferred from neighbouring sites
*wR*(*F*^2^) = 0.155	H atoms treated by a mixture of independent and constrained refinement
*S* = 1.10	*w* = 1/[σ^2^(*F*_o_^2^) + (0.0597*P*)^2^ + 0.8906*P*] where *P* = (*F*_o_^2^ + 2*F*_c_^2^)/3
3151 reflections	(Δ/σ)_max_ = 0.027
274 parameters	Δρ_max_ = 0.87 e Å^−^^3^
2 restraints	Δρ_min_ = −0.58 e Å^−^^3^

### Special details

**Table:**

Geometry. All e.s.d.'s (except the e.s.d. in the dihedral angle between two l.s. planes) are estimated using the full covariance matrix. The cell e.s.d.'s are taken into account individually in the estimation of e.s.d.'s in distances, angles and torsion angles; correlations between e.s.d.'s in cell parameters are only used when they are defined by crystal symmetry. An approximate (isotropic) treatment of cell e.s.d.'s is used for estimating e.s.d.'s involving l.s. planes.
Refinement. Refinement of *F*^2^ against ALL reflections. The weighted *R*-factor *wR* and goodness of fit *S* are based on *F*^2^, conventional *R*-factors *R* are based on *F*, with *F* set to zero for negative *F*^2^. The threshold expression of *F*^2^ > σ(*F*^2^) is used only for calculating *R*-factors(gt) *etc*. and is not relevant to the choice of reflections for refinement. *R*-factors based on *F*^2^ are statistically about twice as large as those based on *F*, and *R*- factors based on ALL data will be even larger.

### Fractional atomic coordinates and isotropic or equivalent isotropic displacement parameters (Å^2^)

**Table:**

	*x*	*y*	*z*	*U*_iso_*/*U*_eq_	
Cu1	0.5000	0.5000	0.5000	0.0217 (3)	
Cu2	0.31791 (11)	0.03642 (9)	0.30349 (7)	0.0197 (3)	
N1	0.5830 (7)	0.3127 (5)	0.4748 (4)	0.0162 (12)	
N2	0.5312 (7)	0.1718 (6)	0.4112 (4)	0.0185 (12)	
N3	0.3647 (7)	0.1167 (6)	0.1569 (4)	0.0200 (13)	
N4	0.1358 (7)	−0.1271 (6)	0.1893 (5)	0.0223 (13)	
O1	0.7315 (6)	0.5766 (5)	0.6067 (4)	0.0232 (11)	
O2	0.9943 (6)	0.5210 (5)	0.6534 (4)	0.0256 (12)	
O3	0.4855 (6)	−0.1054 (5)	0.3024 (4)	0.0190 (11)	
O4	0.7632 (6)	−0.1122 (5)	0.3758 (4)	0.0228 (11)	
O5	0.3188 (6)	0.4290 (5)	0.6487 (4)	0.0301 (12)	
H5A	0.2105	0.4377	0.6272	0.036*	
H5B	0.2736	0.3280	0.6305	0.036*	
O6	0.1384 (8)	0.1048 (7)	0.3818 (5)	0.0413 (15)	
H6A	0.025 (5)	0.049 (8)	0.365 (7)	0.050*	
H6B	0.175 (11)	0.116 (10)	0.453 (2)	0.050*	
O7	0.3725 (8)	0.5886 (6)	0.1884 (5)	0.0453 (16)	
H7A	0.4402	0.5425	0.2201	0.054*	
H7B	0.4142	0.6789	0.2299	0.054*	
C1	0.8372 (9)	0.4887 (7)	0.6033 (5)	0.0168 (14)	
C2	0.7527 (9)	0.3356 (7)	0.5299 (5)	0.0165 (14)	
C3	0.8129 (8)	0.2092 (7)	0.5029 (5)	0.0159 (14)	
H3	0.9272	0.1942	0.5296	0.019*	
C4	0.6679 (8)	0.1085 (7)	0.4275 (5)	0.0147 (14)	
C5	0.6392 (9)	−0.0491 (7)	0.3650 (5)	0.0144 (14)	
C6	0.4736 (10)	0.2407 (8)	0.1428 (7)	0.0294 (18)	
H6	0.5417	0.3154	0.2054	0.035*	
C7	0.4934 (11)	0.2670 (8)	0.0375 (7)	0.0342 (19)	
H7	0.5729	0.3576	0.0299	0.041*	
C8	0.3963 (10)	0.1598 (9)	−0.0520 (6)	0.0305 (18)	
H8	0.4087	0.1752	−0.1228	0.037*	
C9	0.2791 (10)	0.0281 (8)	−0.0407 (6)	0.0253 (17)	
C10	0.1717 (10)	−0.0923 (9)	−0.1297 (6)	0.0296 (18)	
H10	0.1831	−0.0840	−0.2021	0.036*	
C11	0.0548 (10)	−0.2173 (8)	−0.1152 (6)	0.0277 (18)	
H11	−0.0148	−0.2939	−0.1763	0.033*	
C12	0.0373 (9)	−0.2327 (8)	−0.0056 (6)	0.0223 (16)	
C13	−0.0827 (10)	−0.3571 (8)	0.0162 (7)	0.0303 (19)	
H13	−0.1585	−0.4353	−0.0420	0.036*	
C14	−0.0892 (10)	−0.3643 (8)	0.1231 (6)	0.0309 (18)	
H14	−0.1693	−0.4481	0.1393	0.037*	
C15	0.0211 (9)	−0.2496 (8)	0.2065 (6)	0.0247 (16)	
H15	0.0158	−0.2573	0.2797	0.030*	
C16	0.1457 (9)	−0.1181 (8)	0.0838 (6)	0.0221 (16)	
C17	0.2664 (9)	0.0116 (8)	0.0656 (6)	0.0217 (16)	

### Atomic displacement parameters (Å^2^)

**Table:**

	*U*^11^	*U*^22^	*U*^33^	*U*^12^	*U*^13^	*U*^23^
Cu1	0.0237 (7)	0.0135 (6)	0.0264 (8)	0.0099 (5)	−0.0023 (5)	−0.0002 (5)
Cu2	0.0173 (5)	0.0231 (5)	0.0140 (5)	0.0037 (3)	0.0001 (3)	−0.0038 (4)
N1	0.019 (3)	0.007 (3)	0.016 (3)	−0.002 (2)	−0.003 (2)	−0.002 (2)
N2	0.019 (3)	0.020 (3)	0.018 (3)	0.008 (2)	0.002 (2)	0.005 (2)
N3	0.025 (3)	0.020 (3)	0.018 (3)	0.012 (2)	0.004 (2)	0.004 (2)
N4	0.019 (3)	0.025 (3)	0.019 (3)	0.006 (2)	0.002 (2)	−0.003 (3)
O1	0.024 (3)	0.016 (2)	0.028 (3)	0.010 (2)	−0.001 (2)	0.000 (2)
O2	0.023 (3)	0.021 (3)	0.029 (3)	0.009 (2)	−0.003 (2)	−0.001 (2)
O3	0.019 (2)	0.013 (2)	0.022 (3)	0.0055 (19)	−0.002 (2)	0.000 (2)
O4	0.023 (3)	0.021 (2)	0.023 (3)	0.009 (2)	0.003 (2)	0.000 (2)
O5	0.025 (3)	0.022 (3)	0.042 (3)	0.006 (2)	0.004 (2)	0.006 (2)
O6	0.032 (3)	0.047 (4)	0.040 (4)	0.006 (3)	0.004 (3)	0.006 (3)
O7	0.056 (4)	0.027 (3)	0.040 (4)	0.011 (3)	−0.016 (3)	−0.004 (3)
C1	0.020 (4)	0.018 (3)	0.009 (3)	0.004 (3)	−0.002 (3)	0.003 (3)
C2	0.025 (4)	0.010 (3)	0.012 (4)	0.002 (3)	0.003 (3)	0.001 (3)
C3	0.015 (3)	0.017 (3)	0.018 (4)	0.007 (3)	0.001 (3)	0.006 (3)
C4	0.021 (3)	0.015 (3)	0.013 (4)	0.009 (3)	0.010 (3)	0.005 (3)
C5	0.021 (4)	0.012 (3)	0.011 (3)	0.004 (3)	0.004 (3)	0.003 (3)
C6	0.031 (4)	0.023 (4)	0.032 (5)	0.009 (3)	0.003 (3)	0.001 (3)
C7	0.039 (5)	0.023 (4)	0.044 (5)	0.008 (3)	0.011 (4)	0.013 (4)
C8	0.028 (4)	0.046 (5)	0.026 (4)	0.018 (4)	0.013 (3)	0.015 (4)
C9	0.027 (4)	0.034 (4)	0.017 (4)	0.018 (3)	0.005 (3)	0.001 (3)
C10	0.034 (4)	0.049 (5)	0.012 (4)	0.024 (4)	0.005 (3)	0.004 (3)
C11	0.031 (4)	0.025 (4)	0.021 (4)	0.011 (3)	−0.004 (3)	−0.007 (3)
C12	0.025 (4)	0.026 (4)	0.015 (4)	0.016 (3)	−0.002 (3)	−0.005 (3)
C13	0.029 (4)	0.016 (4)	0.038 (5)	0.004 (3)	−0.002 (3)	−0.007 (3)
C14	0.027 (4)	0.028 (4)	0.029 (5)	−0.003 (3)	0.000 (3)	0.003 (3)
C15	0.023 (4)	0.023 (4)	0.027 (4)	0.006 (3)	0.005 (3)	0.003 (3)
C16	0.018 (4)	0.027 (4)	0.021 (4)	0.009 (3)	0.003 (3)	−0.002 (3)
C17	0.024 (4)	0.025 (4)	0.020 (4)	0.015 (3)	0.006 (3)	0.003 (3)

### Geometric parameters (Å, °)

**Table:**

Cu1—O1	1.974 (5)	C1—C2	1.499 (8)
Cu1—O1^i^	1.974 (5)	C2—C3	1.374 (8)
Cu1—N1^i^	1.990 (5)	C3—C4	1.388 (8)
Cu1—N1	1.990 (5)	C3—H3	0.9500
Cu2—N2	1.983 (5)	C4—C5	1.491 (8)
Cu2—O6	1.979 (6)	C6—C7	1.423 (10)
Cu2—N4	2.005 (5)	C6—H6	0.9500
Cu2—O3	2.059 (4)	C7—C8	1.359 (10)
Cu2—N3	2.171 (6)	C7—H7	0.9500
N1—C2	1.343 (8)	C8—C9	1.388 (10)
N1—N2	1.350 (7)	C8—H8	0.9500
N2—C4	1.335 (8)	C9—C17	1.399 (9)
N3—C6	1.318 (9)	C9—C10	1.436 (10)
N3—C17	1.368 (8)	C10—C11	1.357 (10)
N4—C15	1.344 (9)	C10—H10	0.9500
N4—C16	1.368 (8)	C11—C12	1.445 (10)
O1—C1	1.287 (8)	C11—H11	0.9500
O2—C1	1.227 (7)	C12—C13	1.402 (10)
O3—C5	1.266 (7)	C12—C16	1.415 (9)
O4—C5	1.242 (7)	C13—C14	1.377 (10)
O5—H5A	0.8692	C13—H13	0.9500
O5—H5B	0.8987	C14—C15	1.378 (10)
O6—H6A	0.88 (2)	C14—H14	0.9500
O6—H6B	0.88 (2)	C15—H15	0.9500
O7—H7A	0.8526	C16—C17	1.421 (10)
O7—H7B	0.8695		
			
O1—Cu1—O1^i^	180.000 (1)	C4—C3—H3	128.1
O1—Cu1—N1^i^	97.50 (19)	N2—C4—C3	110.0 (5)
O1^i^—Cu1—N1^i^	82.50 (19)	N2—C4—C5	117.1 (6)
O1—Cu1—N1	82.50 (19)	C3—C4—C5	132.9 (6)
O1^i^—Cu1—N1	97.50 (19)	O4—C5—O3	125.9 (6)
N1^i^—Cu1—N1	180.000 (1)	O4—C5—C4	119.6 (6)
N2—Cu2—O6	94.4 (2)	O3—C5—C4	114.5 (5)
N2—Cu2—N4	168.4 (2)	N3—C6—C7	122.6 (7)
O6—Cu2—N4	95.8 (2)	N3—C6—H6	118.7
N2—Cu2—O3	80.9 (2)	C7—C6—H6	118.7
O6—Cu2—O3	142.7 (2)	C8—C7—C6	118.6 (7)
N4—Cu2—O3	87.6 (2)	C8—C7—H7	120.7
N2—Cu2—N3	100.5 (2)	C6—C7—H7	120.7
O6—Cu2—N3	118.2 (2)	C7—C8—C9	120.6 (7)
N4—Cu2—N3	79.4 (2)	C7—C8—H8	119.7
O3—Cu2—N3	98.95 (19)	C9—C8—H8	119.7
C2—N1—N2	107.8 (5)	C8—C9—C17	117.2 (7)
C2—N1—Cu1	111.5 (4)	C8—C9—C10	125.0 (7)
N2—N1—Cu1	140.3 (4)	C17—C9—C10	117.9 (7)
C4—N2—N1	108.1 (5)	C11—C10—C9	123.0 (7)
C4—N2—Cu2	112.9 (4)	C11—C10—H10	118.5
N1—N2—Cu2	139.0 (4)	C9—C10—H10	118.5
C6—N3—C17	117.7 (6)	C10—C11—C12	119.3 (6)
C6—N3—Cu2	131.6 (5)	C10—C11—H11	120.4
C17—N3—Cu2	110.7 (4)	C12—C11—H11	120.4
C15—N4—C16	118.2 (6)	C13—C12—C16	118.4 (7)
C15—N4—Cu2	126.5 (5)	C13—C12—C11	122.7 (6)
C16—N4—Cu2	115.0 (4)	C16—C12—C11	118.9 (6)
C1—O1—Cu1	115.1 (4)	C14—C13—C12	119.1 (7)
C5—O3—Cu2	114.6 (4)	C14—C13—H13	120.5
H5A—O5—H5B	88.9	C12—C13—H13	120.5
Cu2—O6—H6A	119 (5)	C15—C14—C13	119.7 (7)
Cu2—O6—H6B	109 (6)	C15—C14—H14	120.1
H6A—O6—H6B	107 (8)	C13—C14—H14	120.1
H7A—O7—H7B	100.3	N4—C15—C14	123.2 (7)
O2—C1—O1	125.7 (6)	N4—C15—H15	118.4
O2—C1—C2	120.2 (6)	C14—C15—H15	118.4
O1—C1—C2	114.1 (5)	N4—C16—C12	121.5 (6)
N1—C2—C3	110.3 (5)	N4—C16—C17	118.2 (6)
N1—C2—C1	115.9 (5)	C12—C16—C17	120.3 (7)
C3—C2—C1	133.8 (6)	N3—C17—C9	123.3 (6)
C2—C3—C4	103.9 (5)	N3—C17—C16	116.1 (6)
C2—C3—H3	128.1	C9—C17—C16	120.5 (6)

Symmetry codes: (i) −*x*+1, −*y*+1, −*z*+1.

### Hydrogen-bond geometry (Å, °)

**Table:**

*D*—H···*A*	*D*—H	H···*A*	*D*···*A*	*D*—H···*A*
O5—H5A···O2^ii^	0.87	2.05	2.842 (7)	152
O5—H5B···O4^iii^	0.90	1.95	2.824 (7)	163
O6—H6A···O4^ii^	0.88 (5)	2.26 (6)	3.104 (8)	161 (7)
O6—H6B···O4^iii^	0.89 (3)	2.16 (3)	3.042 (8)	172 (10)
O7—H7A···O5^i^	0.85	2.24	2.977 (8)	145
O7—H7B···O3^iv^	0.87	1.97	2.829 (7)	170

Symmetry codes: (ii) *x*−1, *y*, *z*; (iii) −*x*+1, −*y*, −*z*+1; (i) −*x*+1, −*y*+1, −*z*+1; (iv) *x*, *y*+1, *z*.
